# Simulation Game Versus Multiple Choice Questionnaire to Assess the Clinical Competence of Medical Students: Prospective Sequential Trial

**DOI:** 10.2196/23254

**Published:** 2020-12-16

**Authors:** Tristan Fonteneau, Elodie Billion, Cindy Abdoul, Sebastien Le, Alice Hadchouel, David Drummond

**Affiliations:** 1 Department of Paediatric Pulmonology and Allergology University Hospital Necker-Enfants Malades Assistance Publique - Hôpitaux de Paris Paris France; 2 iLumens Simulation Department University of Paris Paris France

**Keywords:** serious game, simulation game, assessment, professional competence, asthma, pediatrics

## Abstract

**Background:**

The use of simulation games (SG) to assess the clinical competence of medical students has been poorly studied.

**Objective:**

The objective of this study was to assess whether an SG better reflects the clinical competence of medical students than a multiple choice questionnaire (MCQ).

**Methods:**

Fifth-year medical students in Paris (France) were included and individually evaluated on a case of pediatric asthma exacerbation using three successive modalities: high-fidelity simulation (HFS), considered the gold standard for the evaluation of clinical competence, the SG *Effic’Asthme*, and an MCQ designed for the study. The primary endpoint was the median kappa coefficient evaluating the correlation of the actions performed by the students between the SG and HFS modalities and the MCQ and HFS modalities. Student satisfaction was also evaluated.

**Results:**

Forty-two students were included. The actions performed by the students were more reproducible between the SG and HFS modalities than between the MCQ and HFS modalities (*P*=.04). Students reported significantly higher satisfaction with the SG (*P*<.01) than with the MCQ modality.

**Conclusions:**

The SG *Effic’Asthme* better reflected the actions performed by medical students during an HFS session than an MCQ on the same asthma exacerbation case. Because SGs allow the assessment of more dimensions of clinical competence than MCQs, they are particularly appropriate for the assessment of medical students on situations involving symptom recognition, prioritization of decisions, and technical skills.

**Trial Registration:**

ClinicalTrials.gov NCT03884114; https://clinicaltrials.gov/ct2/show/NCT03884114

## Introduction

An essential mission of medical schools is to regularly assess the clinical competence of their medical students. These assessments are made difficult by the multidimensional aspects of medical competence, and different methods have been developed [1]. High-fidelity simulation (HFS) and objective structured clinical examination (OSCE) are currently considered the best modalities to assess the clinical competence of medical students because they represent reliable, valid, and acceptable assessment methods without any risk for patients [2-4]. In addition, they allow several dimensions of medical competence to be assessed at the same time, such as knowledge, clinical reasoning, technical skills, and teamwork. However, HFS and OSCE are difficult to implement on a large scale because they require a lot of human resources and faculty time is a scarce resource [5]. In addition, summative assessments with these techniques are suboptimal because of the subjectivity of the evaluators even when a standardized checklist is used and because of the difficulty of proposing the same situations to all medical students, who cannot be evaluated at the same time. Alternatively, multiple choice questionnaires (MCQ) represent a reliable, acceptable, and inexpensive method of assessment that can be widely deployed and offer automated correction. However, MCQs have several drawbacks: they only test knowledge and clinical reasoning, they create situations in which a student can answer a question by recognizing the correct option but would not have been able to answer it in the absence of options (cueing effect), and they limit choices to 4 or 5 proposals when there are many more in real life [4]. There is, therefore, a need for an interim evaluation method, bringing together most of the strengths of the HFS/OSCE and MCQ modalities.

Simulation games (SGs) may represent this solution. SGs combine the features of serious games and simulation. Serious games are defined as games specifically designed for a serious purpose, such as providing health professions education [6]. As serious games, SGs incorporate rules and predefined educational objectives to win the game. This is different from virtual simulators, which can be used without predefined objectives. For example, in Microsoft Flight Simulator, the simulator reproduces the conditions of a real environment but no objective is provided to the player, who can choose the airport they want to fly to [7]. SGs can be defined as a type of serious games designed to closely simulate real-world activities [8]. They belong to the broader group of serious games in that they include preestablished objectives (eg, the patient’s recovery). They share with virtual simulators their realistic, artificial environment in which learners can apprehend the consequences of their decisions.

As serious games, SGs promote attention, active learning, feedback, and consolidation, which have been identified as the four main pillars of learning by cognitive scientists [9]. Moreover, the objective of winning incorporated in SGs enhances learners’ motivation and engagement [7,10]. As simulations, SGs allow users to acquire complex behavioral and technical skills that cannot be entirely acquired through knowledge-based training methods alone and also have the advantage of being risk free for patients and learners [11,12].

SGs have become increasingly popular in the training of health professionals in recent years, and their educational effectiveness has been confirmed by several studies [8,13,14]. However, they have been poorly evaluated as an assessment tool for medical students [15]. Similar to MCQs, SGs offer a standardized assessment that can be given simultaneously on a large scale and inexpensive, automated, and objective correction. They go beyond MCQs by allowing a larger degree of freedom in the options that can be chosen by students and assessment of certain characteristics of the physical examination (inspection, auscultation) and technical skills such as the use of a pressurized metered-dose inhaler with a spacer. However, it is unclear whether an SG is a better method of assessment than a conventional MCQ for evaluating the clinical competence of medical students.

The objective of this study was to compare the performance of an SG and an MCQ to assess the clinical competence of medical students in a scenario of pediatric asthma exacerbation. The gold standard chosen was HFS, and the actions performed on the SG and the responses provided on the MCQ were compared with the actions performed by students during the HFS session to analyze the degree of concordance between the SG and HFS modalities on the one hand and the MCQ and HFS modalities on the other hand.

## Methods

### Ethics

A prospective, simulation-based trial was conducted in the department of simulation in health care iLumens (a multidisciplinary university medical laboratory focused on digital health education) in Paris Descartes University (France). The study was approved by the ethics committee of our institution (CENEM 2019-15-DD) and registered in ClinicalTrials.gov [NCT03884114].

### Participants

Participants were fifth-year medical students from the French faculty of medicine Paris Descartes who participated voluntarily. They were part of two classes and went to the department of simulation in March and June 2019 after their 3-month pediatrics courses which included a 2-hour course on pediatric asthma exacerbation management and within 15 days after their pediatrics exams. Recruitment was done by emails and during their pediatric exams. Written consent was obtained, and demographic data were collected from participants.

### Study Design

**Figure 1:**
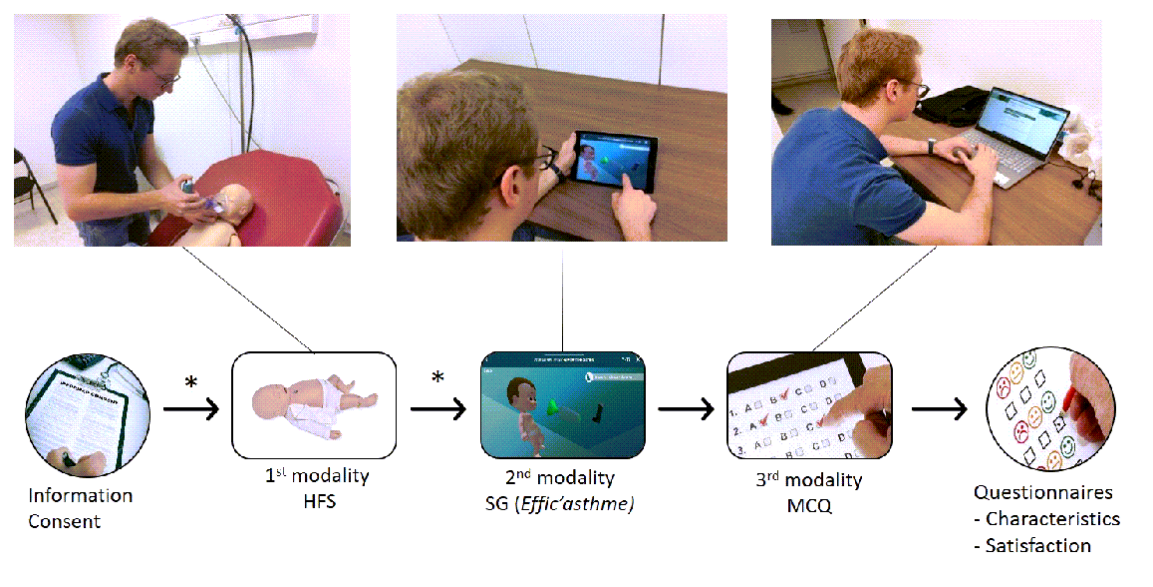
Study design. HFS: high fidelity simulation; SG: simulation game; MCQ: multiple choice questionnaire; *: tutorial video.

The study design is presented in Figure 1. Participants were successively evaluated on the same scenario of a moderate asthma exacerbation occurring in a private medical practice using three different modalities (HFS, SG, and MCQ) for a maximum duration of 12 minutes for each modality. Participants started by watching a 5-minutes tutorial video on the features of the high-fidelity manikin SimBaby (Laerdal Medical AS) used and its environment. They were then individually evaluated on the management of a moderate asthma exacerbation scenario on HFS. No debriefing was provided, and participants were invited to watch a 3-minute video on the features of the SG (*Effic’Asthme*, iLumens, and Dowino). The second evaluation was conducted on the same scenario using the SG. Again, no debriefing was provided, and the third evaluation was conducted on the same scenario using an MCQ. Finally, participants completed a questionnaire on their characteristics, followed by a second questionnaire about their satisfaction with each evaluation modality and ending with an oral debriefing.

### Scenario and Evaluation Modalities

A single scenario was used for all three modalities. It corresponded to a moderate exacerbation of asthma in a child aged 12-months who did not respond to short-acting beta-agonists (SABAs). A full description of the scenario is presented in Multimedia Appendix 1 (Table S1). Participants were expected to administer the emergency treatment (salbutamol, SABAs) to the child with the correct inhalation technique and dose, repeat this administration after no improvement was noted, repeat this administration again adding oral corticosteroids, and finally refer the child to the nearest pediatric emergency department.

**Figure 2:**
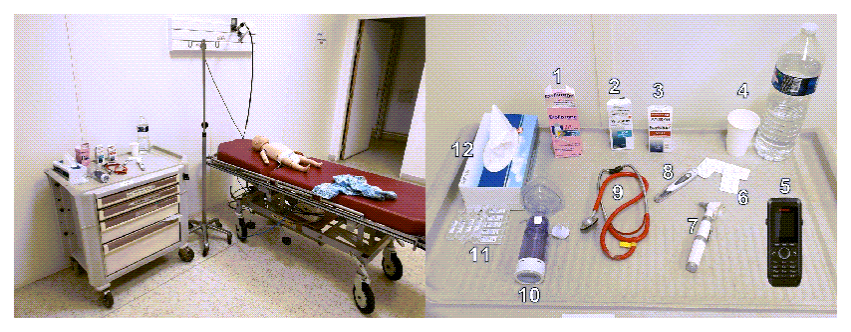
Simulation room with the Simbaby manikin and equipment available to the student: (1) paracetamol, (2) emergency treatment (salbutamol inhaler), (3) controller treatment (fluticasone inhaler), (4) water, (5) telephone to call 911 or pediatric emergencies, (6) oral corticosteroids (prednisolone 20 mg tablets), (7) otoscope, (8) thermometer, (9) stethoscope, (10) inhalation chamber with face mask, (11) saline pipettes, and (12) paper tissue.

The first evaluation modality studied was HFS. As it was impossible to assess the students on their ability to manage an asthma exacerbation with a real patient, we considered HFS the gold standard assessment method that would best reflect students’ clinical competence. The Simbaby manikin used can reproduce many signs of an asthma exacerbation. In the simulation room, participants could use the same items as in the SG (Figure 2): an emergency treatment (salbutamol, SABAs), a controller treatment (fluticasone), an asthma spacer with a facial mask, oral steroids in tablets (prednisone), a glass of water, paracetamol, saline nose drops for nasal airway clearance, and a phone. All actions performed by the participants were video recorded.

**Figure 3:**
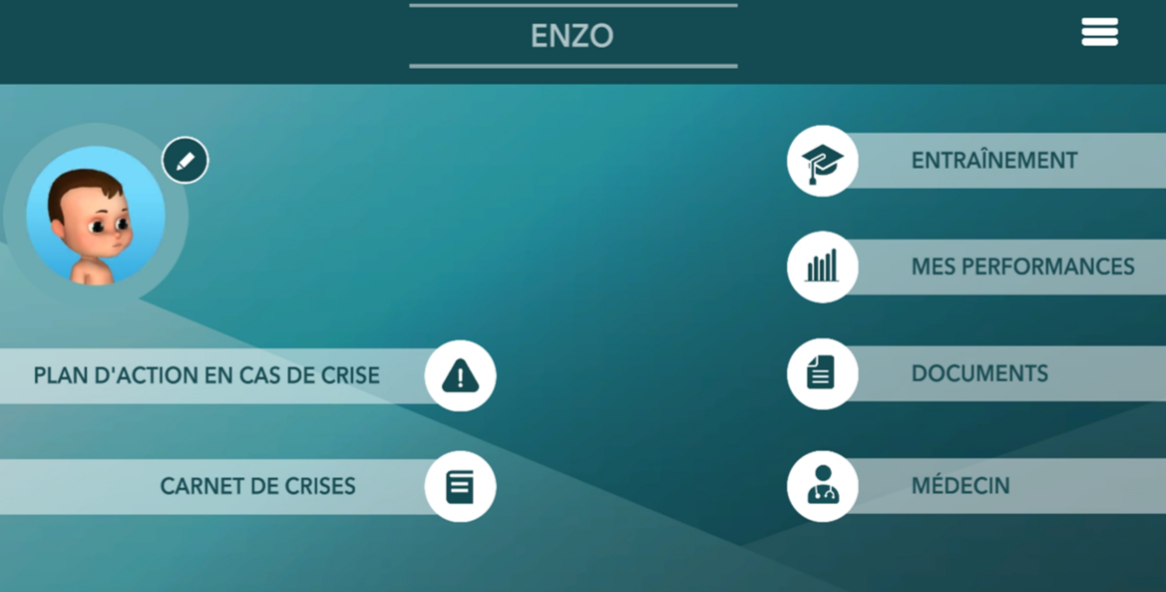
*Effic’Asthme* home screen: (A) “asthma action plan” automatically created by the app based on data (age, weight) entered by parent, (B) “asthma attack log” for monitoring, (C) “training” section, which allows the player to access different scenarios of simulated asthma exacerbation (only this section was used in this study), (D) “my performances,” providing scores on the scenarios already done, (E) “documents,” with access to detailed information on the different asthma symptoms and several questions/answers about the child's asthma, and (F) “physician” section, which helps the treating physician to know which parts of the action plan or inhalation technique should be reviewed with the family.

**Figure 4:**
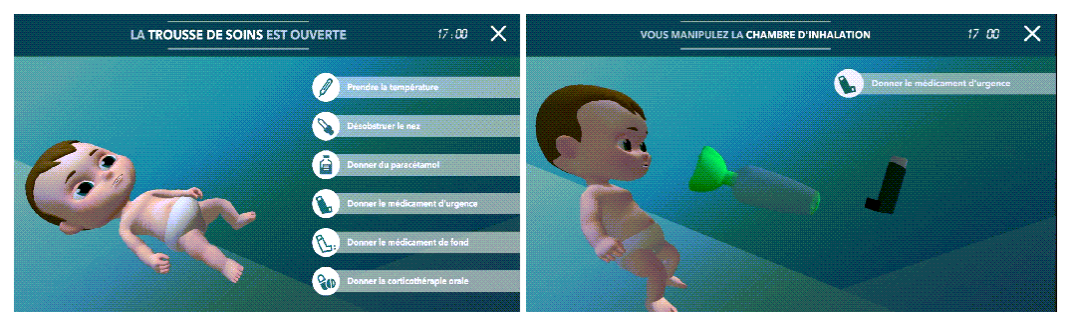
*Effic’Asthme* menu and inhalation technique page: (left) drop-down menu “emergency treatment kit” with several options (take temperature, clean nasal airway, administer paracetamol, administer emergency treatment, administer controller treatment, administer oral corticosteroids) and (right) administration of the inhaler treatment.

The second modality used was the SG *Effic’Asthme*, a mobile app displayed on a tablet. This SG was developed by iLumens at Paris Descartes University and Dowino to train parents to recognize and manage different scenarios of asthma exacerbation in their children [16]. When connecting to *Effic’Asthme* for the first time, the user is asked to enter information to create a child avatar. The home screen then proposes the following sections: asthma action plan, asthma attack log, training, documents, and doctor (Figure 3). The training section corresponds to the SG. After completing a tutorial, the player can choose among 6 scenarios of asthma exacerbations with varying levels of severity, all taking place in the virtual bedroom of the child. A video presenting the main features of *Effic’Asthme* is available (Multimedia Appendix 2). Each scenario starts with a short briefing of the situation. A message then invites the user to carefully observe the child’s avatar with the possibility of zooming and rotating the child in 3D to detect any sign of respiratory distress and to listen for a cough or wheezing. Depending on the child’s condition, the user needs to choose the actions to be performed to manage the asthma exacerbation appropriately among different panels (Figure 4 left). One of the main outcomes is to check the administration technique of the emergency treatment (salbutamol/albuterol) to the child (Figure 4 right). The user can use his/her fingers to remove the cap of the spray, shake it, insert it into the spacer, place the spacer on the child’s face, press the spray to administer one puff, and wait for the number of breaths they deem necessary between each puff. Once the scenario is completed, an automated, point-by-point debriefing is provided. Points are awarded for actions performed correctly, and an overall success rate of the mission out of 100% is given.

For the study, *Effic’Asthme* was diverted from its initial use to assess students’ clinical competence. This SG was chosen because it allowed the evaluation of several dimensions of clinical competence such as knowledge and clinical reasoning and technical skills such as the administration technique of the inhaled emergency treatment.

The third assessment modality was an MCQ designed for the study (Table S2 in Multimedia Appendix 1). As with the HFS and SG, the MCQ started with a short briefing followed by 15 questions on the management of the same scenario of moderate asthma exacerbation.

### Data Collection and Outcomes

For each evaluation modality, the investigators completed a standardized checklist (common for the three modalities, Table S3 in Multimedia Appendix 1). This checklist had been previously validated [16,17]. The checklist included 19 items. Each item was rated 0 or 1, depending on whether or not the student had performed the correct action when evaluated on the HFS or SG or provided the correct answer on the MCQ, with a maximum score of 19.

The main outcome was the degree of correlation between the actions performed for each item between the SG and HFS on the one hand and the concordance of the answers on the MCQ and the actions performed on the HFS on the other hand. For each item rated 0 or 1 for the modalities SG and HFS or MCQ and HFS, the Cohen kappa coefficient was calculated to estimate the concordance of the actions performed and answers provided at the level of the population studied. The median kappa coefficients on the 19 items were calculated and compared between the SG/HFS group and the MCQ/HFS group.

Secondary outcomes included the comparison between the median checklist scores for the HFS, SG, and MCQ modalities; comparison of the dispersion of the checklist scores for each modality; and participant satisfaction with the three assessment modalities.

### Statistical Analysis

The sample size calculation was based on the following assumptions: 2-tailed alpha of .05, power of 80%, a median kappa coefficient estimated at 0.4 for the MCQ/HFS comparisons, and an expected median kappa coefficient of 0.7 for the SG/HFS comparisons. The calculation suggested that at least 39 participants should be enrolled [18].

Data were collected in Excel (Microsoft Corp) and analyzed with Prism software version 5.03 (GraphPad). Median values and interquartile ranges were reported. Cohen kappa coefficients for each item of the checklist between the HFS/SG and HFS/MCQ groups were calculated. The median kappa coefficients for each group were compared using a Mann-Whitney *U* test to compare the correlation of actions and answers between these two groups. The Spearman rank correlation coefficient was used to compare the median checklist scores between the three modalities. Finally, the dispersion of scores between the SG and MCQ modalities was studied by comparing variances using a modified Levene test, and participant satisfaction for each modality was compared using a student *t* test.

## Results

A total of 42 students were included in the study in March and June 2019. Their characteristics are presented in Table 1. No participant was lost to follow-up.

**Table 1:** Participant characteristics.

Characteristics				Value (n=42)
**General**				
	Female, n (%)		28 (67)	
	Age in years, median (interquartiles 1,3)		24 (23-24)	
**Asthma experience**				
	**Personal history of asthma, n (%)**			
		Current asthma	1 (2)	
		Asthma during childhood	4 (10)	
		No history of asthma	37 (88)	
	Internship in general pediatrics, pediatric emergency, and/or pediatric pneumology over the past 24 months, n (%)		14 (33)	
	Ever witnessed an asthma attack, n (%)		7 (17)	
**Simulation experience, n (%)**				
	High-fidelity simulation experience		41 (98)	
	Experience with the Simbaby high-fidelity manikin		11 (26)	
**Video games experience, n (%)**				
	Ever played video games		37 (88)	
	Played video games in the past 6 months		15 (36)	
	**Frequency of play, n (%)**			
		Never	11 (26)	
		Less than once a month	24 (57)	
		Several times a month	6 (14)	
		Several times a week	0 (0)	
		Every day or almost every day	1 (3)	
	**Equipment owned, n (%)**			
		Smartphone	42 (100)	
		Computer	42 (100)	
		Digital tablet	16 (38)	
		Game console	18 (43)	

**Figure 5:**
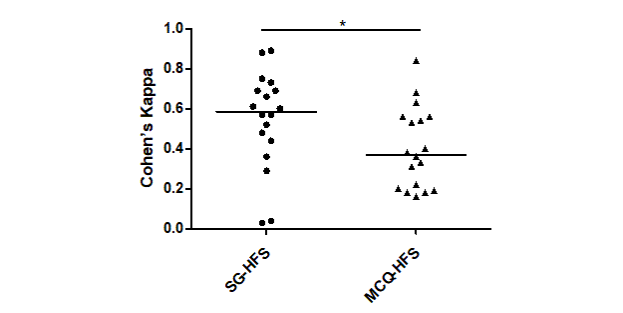
Concordance of actions between the modalities assessed by Cohen kappa coefficient. Each point represents the median degree of agreement (Cohen kappa coefficient) of all students for one of the 19 items on the checklist. The horizontal bar corresponds to the median kappa coefficient for all items. HFS: high-fidelity simulation; SG: simulation game; MCQ: multiple choice questionnaire; *P=.04.

The concordance of the actions performed during the SG session and the HFS session was moderate [19], with a median Cohen kappa coefficient (interquartile [IQ] 1-3) of 0.59 (IQ 0.45-0.69; Figure 5). The concordance of the answers provided in the MCQ and the actions performed during the HFS session was weak, with a median Cohen kappa coefficient of 0.37 (IQ 0.20-0.56). The SG offered a higher level of concordance with the actions performed during the HFS session than the MCQ as attested by the comparison of the median Cohen kappa coefficient (*P*=.04).

**Figure 6:**
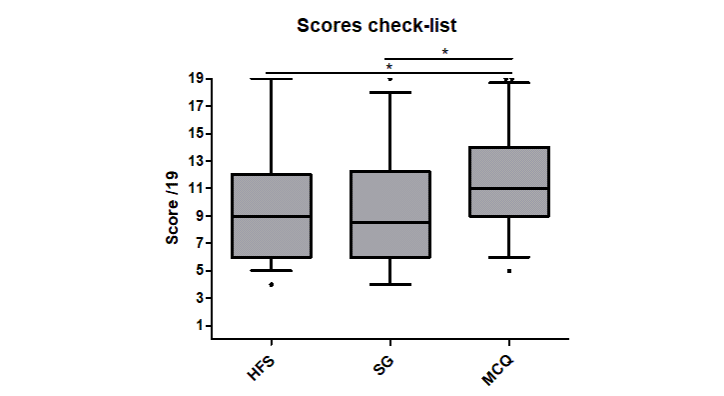
Median checklist scores for the modalities. HFS: high-fidelity simulation; SG: simulation game; MCQ: multiple choice questionnaire; *P=.04.

The median checklist scores were 9 (IQ 6-12), 8.5 (IQ 6-12), and 11 (IQ 9-14) for the HFS, SG, and MCQ modalities, respectively (Figure 6). These scores were significantly different (*P*=.01), and multiple comparisons found a significantly higher score in the MCQ group than in the HFS (*P*<.05) and SG (*P*<.05) groups.

The correlations between the SG and HFS modalities and the MCQ and HFS modalities were studied using their total checklist scores: the Spearman coefficient was 0.79 (95% CI 0.63-0.88) between the SG/HFS modalities and 0.70 (95% CI 0.50-0.83) between the MCQ/HFS modalities. This was not significantly different (*P*=.11).

The dispersion of the scores measured by the variance (σ2) of the checklist scores (17.1 and 10.9 for SG and MCQ, respectively) was not statistically significant.

Students preferred the SG modality over the MCQ (Table 2). In their opinion, the SG was more effective for progressing in the management of a clinical situation, more representative of their clinical skills, closer to real life, and more fun than MCQ (Table 2). Two-thirds of the students stated that they would prefer to be assessed in the future using SG rather than MCQ.

**Table 2:** Students’ views of the simulation game as an educational tool in medical school.

Questions		Value, n (%)	Value, mean (SD)	*P* value
**I liked this evaluation modality^a^**		—^b^	—	<.001
	SG^c^	—	4.5 (0.6)	—
	MCQ^d^	—	3.7 (1.1)	—
**I felt stress during the evaluation^a^**		—	—	.26
	SG	—	2.6 (1.4)	—
	MCQ	—	2.2 (1.6)	—
**I had fun during the evaluation^a^**		—	—	<.001
	SG	—	3.9 (0.8)	—
	MCQ	—	1.9 (1.5)	—
**I would say that the most effective modality to progress in the management of a clinical situation such as a child asthma exacerbation is...**				
	SG	28 (67)	—	—
	MCQ	14 (33)	—	—
**I would say that the modality most representative of reality is...**				
	SG	41 (98)	—	—
	MCQ	1 (2)	—	—
**I would say that the modality most representative of my skills is...**				
	SG	38 (90)	—	—
	MCQ	4 (10)	—	—
**I would personally prefer to be evaluated in the future by...**				
	SG	27 (64)	—	—
	MCQ	15 (36)	—	—

^a^Results are expressed as a mean (SD) between 1 (strongly disagree) and 5 (strongly agree).

^b^Not applicable.

^c^SG: simulation game.

^d^MCQ: multiple choice questionnaire.

## Discussion

### Principal Findings

HFS and OSCE, the best available modalities for assessing the clinical competence of medical students without any risk to the patient, are almost impossible to implement on a large scale due to their high consumption of time and human resources. Thus, many faculties use MCQ as a default solution for large-scale examinations, with the risk of favoring students who are highly competent to respond to MCQs but unable to manage real-life emergency situations. This study demonstrates that *Effic’Asthme*, an SG created to teach about the management of pediatric asthma exacerbations, better reflects the actions of fifth-year medical students on HFS than an MCQ on the same clinical case. We propose in Table 3 a summary of the domains of clinical competence that can be assessed through an SG such as *Effic’Asthme*, in comparison with MCQ and HFS. As illustrated, if further studies confirm these findings, SG may become an intermediate solution between HFS and MCQ, evaluating students’ clinical competence further than MCQ while remaining widely deployable, unlike HFS.

**Table 3:** Modalities of assessment and domains covered.

Domain	Multiple choice questionnaires	Simulation game	High-fidelity simulation
Knowledge (eg, corticosteroids dose)	+++^a^	**+++** Possible with *Effic’Asthme*	+++
Clinical reasoning (eg, choosing between repeating salbutamol or calling emergency medical services after 20 minutes)	++	**+++** Possible with *Effic’Asthme*	+++
Technical skills (eg, inhalation technique when using a spacer)	–^b^	+ Possible with *Effic’Asthme*	+++
Recognition of symptoms (eg, chest-indrawing, audible wheezing)	–	+++ Possible with *Effic’Asthme*	+++
Communication skills (eg, reassuring the parent)	–	– Not possible with *Effic’Asthme*	++
Teamwork (eg, coordinating with a nurse)	–	++ (multiplayer) Not possible with *Effic’Asthme*	+++

^a^Appropriate to evaluate the domain.

^b^Not appropriate to evaluate the domain.

Contextualizing our results within the medical literature is limited by the paucity of data on the comparison of SG with other assessment modalities. To our knowledge, only the study by Adjedj et al [15] compared the SG and MCQ modalities for the evaluation of medical students, on a clinical case of cardiology. They found greater variability in the scores obtained on the SG than in the scores obtained on the MCQ and concluded that SG had the potential to better rank students [15]. However, because their study did not use a gold standard as ours did, they were unable to determine which of the two assessment modalities was more relevant for evaluating students. Therefore, our study goes further in showing that SG is indeed a more relevant assessment modality than MCQ for the evaluation of the management of a child’s asthma exacerbation, as it better reflects the actions performed on HFS. Surprisingly, whereas the analysis, item by item, of the actions performed/answers provided revealed a higher level of concordance between the SG/HFS modalities than between the MCQ/HFS modalities, this difference was no longer statistically significant when the total checklist scores were compared (*P*=.11). The most likely explanation is a lack of power because our sample size was not calculated on this outcome.

After considering the SG *Effic’Asthme* as a new evaluation modality, its acceptability and cost should be scrutinized. The results of the survey conducted with the medical students who participated in this study indicate that students support the use of this evaluation modality for their exams, as reported in other studies [20,21]. It would be interesting to collect the opinions of the members of the faculty to see if they are consistent with those of students. Clearly, faculty members will need to estimate the costs of this new assessment modality. Developing SG is costly: their development is costly. For example, the development of *Effic’Asthme* cost €135,000 (US $160,000) [22]. This cost could be reduced in the future by using an interuniversity platform integrating all the necessary elements to create a multitude of scenarios in the same way that high-fidelity simulators are provided with a software allowing to design hundreds of different situations.

A limitation of the SG revealed by this study was the need to integrate a tutorial. Indeed, in our study, many participants did not perform actions such as shaking the inhaler or removing its cap, although they had performed these actions on the HFS and the MCQ. This suggests that they thought it was impossible to perform these actions on the SG. Thus, based on the results of this study, a key recommendation for future developers of SGs is to develop a neutral tutorial before the assessment part of their SG.

### Limitations

The study has several limitations. First, it can be argued that the order of assessments (HFS then SG then MCQ) may have favored higher scores on the MCQ modality. Initially, we considered a crossover trial: after their evaluation with the HFS modality, students would have been randomized into two groups, one group starting by the SG session and ending with the MCQ modality as in this study, and the other starting by the MCQ modality and ending with the SG session. However, the MCQ required students to be provided several cues (eg, when asking for the dose of SABAs or oral corticosteroids, it was implied that SABAs and oral corticosteroids should be administered). This would have strongly biased the next evaluation on SG. By contrast, the HFS and SG modalities did not influence the student on specific actions to be taken because no indication was provided during or after the sessions, and we judged that there was no reason for students to perform better over the course of the different assessments without being provided any indication between them. Indeed, it was demonstrated that no gain in knowledge can be achieved when no debriefing is provided following a simulation session [23]. The use of HFS as a gold standard for assessing medical students’ competence is also debatable. HFS is probably not a modality that is perfectly representative of the care that would have been provided by the students under real-world conditions, but it corresponded to the only option that we have found to be both feasible and ethical. Finally, the fact that the study was monocentric and evaluated only volunteer students on a single scenario limits the generalizability of the results. Despite these limitations, this trial is the first to compare SG and MCQ as assessment methods for medical students.

### Conclusion

In conclusion, the SG used in this study better reflected the clinical competence of students on HFS than an MCQ on the same clinical case of pediatric asthma exacerbation. Its use as an assessment method was appreciated by the students. If further studies confirm these results, SG could become an interesting compromise for the evaluation of medical students between the cheap but limited assessment allowed by MCQs and the comprehensive but highly expensive assessment allowed by HFS.

## Appendix

Multimedia Appendix 1 Supplementary material.

Multimedia Appendix 2 Example of a scenario in *Effic'Asthme*.

## Abbreviations

HFS: high-fidelity simulation
IQ: interquartile
MCQ: multiple choice questionnaires
OSCE: objective structured clinical examination
SABA: short-acting beta-agonist
SG: simulation game
