# Supplementary material for: Simulation Game Versus Multiple Choice Questionnaire to Assess the Clinical Competence of Medical Students: Prospective Sequential Trial
Source: J Med Internet Res. 2020 Dec 16;22(12):e23254. doi: 10.2196/23254 (PMC7773513; doi:10.2196/23254)
Supplement: Multimedia Appendix 1 [file jmir_v22i12e23254_app1.docx]

**Supplementary material**

**Simulation game versus multiple-choice questionnaire to assess the clinical competence of medical students**

**Table S1: Scenario presented at the beginning of the three modalities**

It's 5:00 p.m., you're a doctor doing home visits. You see Enzo who is 12-month-old. His mother speaks little French but you understand she left Enzo at a friend's house all afternoon. When she picked him up, she found that he had difficulty breathing and coughed a lot. In the health record, you note that Enzo has an infant asthma on Fluticasone 50μg 2 puffs twice a day.

**Table S2 : multiple choice questionnaire.**

It's 5:00 p.m., you're a doctor doing home visits. You see Enzo who is 12-month-old. His mother speaks little French, but you understand she left Enzo at a friend's house all afternoon. When she picked him up, she found that he had difficulty breathing and coughed a lot. In the health record, you note that Enzo has an infant asthma on Fluticasone 50μg 2 puffs twice a day.

On clinical examination, Enzo weights 10 kg. The temperature is 37.7°C, he is pink. He has a respiratory rate at 40 breaths per minute, intercostal and subcostal retractions and a seesaw respiration. There is no cyanosis. You hear expiratory wheezing without even using your stethoscope.

*Correct answer(s) are in bold*

For each of the questions below, choose the correct(s) answer(s):

1) Enzo:

a. Does not have an asthma exacerbation

b. Presents a mild asthma exacerbation

**c. Presents a moderate asthma exacerbation**

d. Presents a severe asthma exacerbation

e. Presents an immediate life-threatening asthma exacerbation

2) What is your immediate care?

a. Give paracetamol

b. Give controller treatment (Fluticasone)

**c. Give emergency treatment (Salbutamol)**

d. Give oral corticosteroids (Prednisolone)

e. Call 911 or address Enzo to emergencies

3) You decide to start by giving the emergency treatment in the inhalation chamber: How many puff(s) do you give?

a. 1 puff

b. 2 puffs

**c. 5 puffs**

d. 8 puffs

e. 10 puffs

4) Regarding the inhalation technique

a. The puffs should be administered directly into the child's mouth while pinching his nose.

**b. It is necessary to shake the emergency treatment spray before giving the puffs**

**c. Remove the cap from the emergency treatment spray**

d. Shake the inhalation chamber after each puff

**e. The emergency treatment puffs must be administered once the mask of the inhalation chamber is on the child's face**

5) Regarding the inhalation technique

a. Total number of puffs should be administered at one time and then wait for 1 breath.

b. Total number of puffs should be administered at one time and then wait for 5 breaths

c. After each puff given, wait for 2 breaths

**d. After each puff given, wait for 5 breaths**

e. Giving the total number of puffs at once or each puff separately is equivalent.

6) You give emergency treatment puffs in the inhalation chamber. Thirty seconds later, Enzo keeps couching and wheezing, the respiratory rate and retraction signs are unchanged. What do you decide?

at. Call 911 or address Enzo to emergencies

b. Immediately give again the same number of puffs of the emergency treatment

**c. Wait for treatment to take effect**

d. Give oral corticosteroid therapy.

e. Write the exacerbation treatment prescription and leave the house

7) You decide to wait for the treatment to take effect. How long do you wait?

a. 5 minutes

b. 10 minutes

**c. 20 minutes**

d. 30 minutes

e. 60 minutes

8) After waiting the number of minutes previously chosen, the clinical state is unmodified. What do you do?

**a. Give emergency treatment in the inhalation chamber**

b. Give controller treatment

c. Give oral corticosteroids

d. Give paracetamol

e. Call 911 or address Enzo to emergencies

9) You decide to give the emergency treatment again. How many puff(s) do you give this time?

a. 1 puff

b. 2 puffs

**c. 5 puffs**

d. 8 puffs

e. 10 puffs

10) After giving the emergency treatment, what do you do next?

a. Call 911 or address Enzo to emergencies

**b. Wait for the treatment to take effect**

c. Give oral corticosteroids

d. Give controller treatment

e. Write the exacerbation treatment prescription and leave the house.

11) You decide to wait for the treatment to take effect. How long do you wait this time?

a. 5 min.

b. 10 min.

**c. 20 min.**

d. 30 min.

e. 60 min.

12) The clinical state is unchanged, what do you do?

a. Call 911 or address Enzo to emergencies

**b. Give emergency treatment in the inhalation chamber**

c. Give oral corticosteroids.

d. Write the exacerbation treatment prescription and leave the house.

e. Give the controller treatment

13) You decide to give the emergency treatment again. How many puff(s) do you give this time?

a. 1 puff

b. 2 puffs

**c. 5 puffs**

d. 8 puffs

e. 10 puffs

14) You have waited for the treatment to take effect. It has been one hour since you started the treatment. The clinical examination is still unchanged. What do you do?

**a. Call 911 or address Enzo to emergencies**

**b. Give emergency treatment in the inhalation chamber**

**c. Give oral corticosteroids**

d. Write the exacerbation treatment prescription and leave the house

e. Give the controller treatment

15) You decide to give oral corticosteroid (Presnisolone), what dose do you give?

a. 15 mg

**b. 20 mg**

c. 25 mg

d. 30 mg

e. 35 mg

You call the 911 that arrives 10 minutes later. Enzo is transferred to pediatric emergencies.

**Table S3: Standardized checklist used**

1) SABA (short acting beta-agonist) N°1 given: YES / NO

2) SABA N°1 correct dose (5 puffs): YES / NO

3) Technique: cap removed: YES / NO

4) Technique: inhaler shaked: YES / NO

5) Technique: inhalation chamber well placed on the face of the child: YES / NO

6) Technique: 5 breaths between each puff: YES / NO

7) SABA No. 1: waiting after SABA administration: YES / NO

8) SABA N°1: waiting 20 minutes: YES / NO

9) SABA N°2 given: YES / NO

10) SABA N°2 correct dose (5 puffs): YES / NO

11) SABA N°2: waiting after SABA administration: YES / NO

12) SABA N°2: wait 20 minutes: YES / NO

13) SABA N°3 given: YES / NO

14) SABA N°3 correct dose: YES / NO

15) Emergency transfer or 911 call: YES / NO

16) Emergency transfer or 911 call at the right time (after 3 administrations of SABA): YES / NO

17) Oral corticosteroid given: YES / NO

18) Oral corticosteroid given at the right time (after 3 administrations of SABA): YES / NO

19) Oral corticosteroids: correct dose (20 mg): YES / NO
